# Advanced Glycation End Products and Inflammatory Cytokine Profiles in Maintenance Hemodialysis Patients After the Ingestion of a Protein-Dense Meal

**DOI:** 10.1053/j.jrn.2021.11.006

**Published:** 2021-12-17

**Authors:** Ryan K. Perkins, Stephan van Vliet, Edwin R. Miranda, Kelly N. Z. Fuller, Paul J. Beisswenger, Kenneth R. Wilund, Scott A. Paluska, Nicholas A. Burd, Jacob M. Haus

**Affiliations:** * School of Kinesiology, University of Michigan, Ann Arbor, Michigan.; † Department of Kinesiology and Community Health, University of Illinois at Urbana-Champaign, Urbana, Illinois.; ‡ Kansas University Medical Center, Kansas City, Kansas.; § Geisel School of Medicine, Dartmouth College, Lebanon, New Hampshire.; ¶ Division of Nutritional Sciences, University of Illinois at Urbana-Champaign, Urbana, Illinois.; ** Department of Family Medicine, University of Illinois at Urbana-Champaign, Urbana, Illinois.

**Keywords:** Maintenance Hemodialysis, Inflammation, AGEs, Diet

## Abstract

**Objective:**

The goal of this investigation was to evaluate circulating and skeletal muscle inflammatory biomarkers between maintenance hemodialysis (MHD) and demographic-matched control subjects (CON) before and after ingestion of a protein-rich meal.

**Design and Methods:**

CON (n = 8; 50 ± 2 years; 31 ± 1 kg/m^2^) and MHD patients (n = 8; 56 ± 5 years; 32 ± 2 kg/m^2^) underwent a basal blood draw and muscle biopsy and serial blood draws after the ingestion of a mixed meal on a nondialysis day. Plasma advanced glycation end products (AGEs) and markers of oxidation were assessed via liquid chromatography–tandem mass spectrometry before and after the meal (+240 min). Circulating inflammatory cytokines and soluble receptors for AGE (sRAGE) isoforms (endogenous secretory RAGEs and cleaved RAGEs) were determined before and after the meal (+240 min). Basal muscle was probed for inflammatory cytokines and protein expression of related signaling components (RAGE, Toll-like receptor 4, oligosaccharyltransferase subunit 48, TIR-domain–containing adapter-inducing interferon-*β*, total I*κ*B*α*, and pI*κ*B*α*).

**Results:**

Basal circulating AGEs were 7- to 343-fold higher (*P* < .001) in MHD than those in CON, but only MG-H1 increased in CON after the meal (*P* < .001). There was a group effect (MHD > CON) for total sRAGEs (*P* = .02) and endogenous secretory RAGEs (*P* < .001) and a trend for cleaved RAGEs (*P*=.09), with no meal effect. In addition, there was a group effect (MHD < CON; *P* < .05) for circulating fractalkine, interleukin (IL)10, IL17A, and IL1*β* and a trend (*P* < .10) for IL6 and macrophage inflammatory protein 1 alpha, whereas tumor necrosis factor alpha was higher in MHD (*P* < .001). In muscle, Toll-like receptor 4 (*P* = .03), TIR-domain–containing adapter-inducing interferon-*β* (*P* = .002), and oligosaccharyltransferase subunit 48 (*P* = .02) expression was lower in MHD than that in CON, whereas IL6 was higher (*P* = .01) and IL8 (*P* = .08) tended to be higher in MHD.

**Conclusion:**

Overall, MHD exhibited an exaggerated, circulating, and skeletal muscle inflammatory biomarker environment, and the meal did not appreciably affect the inflammatory status.

## Introduction

THE LOSS OF muscle mass in patients undergoing maintenance hemodialysis (MHD) is well-documented and reported to be a primary consequence of a proinflammatory state.^1-3^ Chronically elevated circulating advanced glycation end products (AGEs) are a major driver of inflammation in patients with chronic kidney disease (CKD),^4-8^ and progression along the CKD continuum is related to increased circulating AGE load.^8,9^ AGEs are a heterogenous group of molecules formed from the nonenzymatic reactions between glucose, or reactive *α*-dicarbonyls such as methylglyoxal, and positively charged amino acid residues.^10-12^ AGEs encourage proinflammatory events through recognition by the receptor for AGEs (RAGE) and subsequent downstream signaling pathway activation (e.g., nuclear-factor-kappa B [NF*κ*B]).^7,13-16^ This ligand-receptor system can be disrupted through the proteolytic release of RAGE, forming a cleaved RAGE isoform (cRAGE)^17^ and alternative RAGE splicing, leading to the production and cellular expulsion of endogenous secretory RAGE (esRAGE).^18^ cRAGE and esRAGE (total sRAGE) suppress AGE-RAGE signaling by (1) reducing signaling potential at the cell due to fewer RAGE receptors and (2) acting as a decoy through binding and sequestering circulating AGEs.^7^

Inflammation disrupts protein homeostasis in MHD, promoting a catabolic environment.^19-21^ In support of this link between inflammation and proteostasis in MHD, past efforts have shown a relationship between release of the proinflammatory cytokine interleukin-6 (IL6) from peripheral tissues and accelerated muscle protein loss.^21^ Furthermore, markers of circulating inflammation, such as c-reactive protein (CRP), are strongly related to indirect measurements of skeletal muscle protein synthesis, degradation, and net protein balance in MHD patients.^19^ Proinflammatory cytokines exert their effects on protein balance by activating downstream proteolytic signaling pathways, including the ubiquitin-proteasome pathway and caspase-3. We recently demonstrated that individuals undergoing MHD have higher basal caspase-3 protein content in skeletal muscle.^22^ Activation of pathways that regulate protein breakdown rates leads to the excess release of amino acids into the muscle intracellular free pool, resulting in elevated muscle protein synthesis rates in the basal state.^23^ This overstimulation of MHD muscle results in anabolic resistance of muscle protein synthesis rates to dietary protein and, over time, likely contributes to accelerated losses in muscle mass in individuals on MHD.^23^

Moreover, protein-energy wasting (i.e., depletion of body protein stores) is another hallmark of advanced CKD and likely contributes to the progression of muscle loss with MHD. Therefore, it is not surprising that increasing the protein density of dietary patterns has received much attention because of the impact of renal replacement therapy on appetite and nutrient availability.^20,24-27^ Evidence exists showing MHD treatment is catabolic in nature,^28^ an outcome potentially driven by modulation of genes involved with apoptosis and inflammation in skeletal muscle^29^ and loss of amino acids in the dialysate.^30^ Coupled with the upregulation of inflammatory signaling pathways in muscle, loss of amino acids during MHD highlights the importance of consuming protein-rich meals between dialysis treatments to counteract the catabolic nature of MHD.

Despite continued illumination of the underlying mechanisms promoting inflammation, our understanding of the inflammatory phenotype in MHD is still limited. It is clear that patients on MHD experience muscle wasting, a condition suspected to be perpetuated by chronic basal inflammation and poor nutrient status. A more complete understanding of the inflammatory burden in patients undergoing MHD will direct targeted therapeutics. Thus, the goal of this investigation was to evaluate the spatial and temporal dynamics of inflammation and inflammatory regulators by evaluating a host of biomarkers in the circulation and skeletal muscle in the basal state and following a mixed, protein-dense meal recommended by the National Kidney Foundation for an MHD population^31^ and a demographic-matched control group. Furthermore, we aimed to build on our previous work and explore a potential relationship between the inflammatory milieu and anabolic resistance to feeding observed in this MHD cohort.^22^

## Methods

### Experimental Design

Recruitment methods for this pilot study have been previously reported, representing a sub-analysis of this work.^22^ Briefly, volunteers were determined to be eligible based on responses to a detailed medical screening questionnaire and blood panel findings. In addition, MHD participants received clearance to participate in the study from their personal nephrologist. All participants were informed about the experimental procedures and potential risks before providing written consent. The study was approved by the Institutional Review Board at the University of Illinois at Urbana-Champagne and conformed to standards for the use of human participants in research as outlined in the Declaration of Helsinki. Clinical Trial Registration: (www.clincialtrials.gov/).

### Participants

MHD (n = 8; 56 ± 5 years) and CON (n = 8; 50 ± 2 years) participants were matched for age, sex, body mass index, and homeostatic model of assessment for insulin resistance (Table 1). MHD participants were taking the following medications: phosphatase binders (n = 8), calcimimetics (n = 8), angiotensin-converting enzyme inhibitors (n = 6), nonsteroidal anti-inflammatory drugs (n = 6), *β*-blockers (n = 4), calcium channel blockers (n = 4), diuretics (n = 4), statins (n = 4), proton pump inhibitors (n = 3), xanthine oxidase inhibitors (n = 3), opioids (n = 3), vasodilators (n = 2), P2Y12 inhibitors (n = 2), serotonin-reuptake inhibitors (n = 2), antihistamine (n = 1), prokinetic (n = 1), and immunosuppressant (n = 1). CON participants were taking nonsteroidal anti-inflammatory drugs (n = 2). Body weight and height were assessed by standard procedures as well as body composition via dual-energy X-ray absorptiometry scan.

### Protocol

A detailed description of the protocol has been described elsewhere.^22^ Briefly, all participants were instructed to refrain from vigorous physical activity for 3 days before the trial and maintain their normal dietary pattern and prescribed medications. Subjects reported to the laboratory at 7:00 AM after consuming a 320 kcal standardized meal (22 g protein, 43 g carbohydrate, and 7 g fat) the night before. Other than this standardized meal, participants were instructed to remain fasted. MHD patients were studied ~24 h after their previous dialysis treatment. The study protocol consisted of a basal blood draw and skeletal muscle biopsy followed by serial blood draws (30, 60, 90, 120, 180, 240, and 300 minutes) after a mixed meal challenge. Circulating postmeal inflammatory assessments were made at the 240-minute time point. Coupled with sample availability, our intention was to capture the inflammatory load in both the postabsorptive and postprandial states. In addition, all participants performed a 2-day dietary recall (Nutritionist Pro v2.1.13, Axxya Systems, Redmond, WA). On average, protein intake tended to be lower (*p* < .10) in MHD than that in CON (MHD: 0.9 ± 0.14; CON: 1.14 ± 0.05 g/kg body weight). No differences in other macronutrients were observed (*p* > .05).

### Mixed Meal Challenge

After basal measurements, each participant consumed a mixed meal consisting of 3 scrambled eggs, 1 slice of toasted white bread, 300 mL apple juice, and 10 g cow butter (total: 546 kcal, 20 g protein, 59 g carbohydrate, and 26 g fat). This meal was selected because it is representative of a standard meal recommended by the National Kidney Foundation for this patient population.^31^ Macronutrient composition of the eggs was determined by the combustion method (method 990.03; AOAC International, 2000; TruMac; LEO Corp., Sain Joseph, MI). For the other foods, macronutrient composition was determined from their respective food labels.

### Blood Draws and Skeletal Muscle Biopsy

A catheter was inserted into a dorsal hand vein and heated for sampling of arterialized venous blood. A skeletal muscle biopsy^32-35^ of the *m.* vastus lateralis was obtained in the basal state under local anesthetic (Lidocaine, 2%). After the muscle biopsy, excess blood, visible fat, and connective tissue were removed and the sample was immediately flash-frozen in liquid nitrogen and stored at −80°C until analysis.

### Blood Analyses

Metabolites (glucose, albumin, creatinine, estimated glomerular filtrate rate) were assessed using a point-of-care chemistry analyzer (Piccolo Xpress Chemistry Analyzer; Abaxis, Union City, CA). Basal plasma insulin and high-sensitivity CRP concentrations were determined using a commercially available enzyme-linked immunosorbent assay (ELISA; Alpco Diagnostics; Salem, NH), and nonesterified fatty acids (basal, 30, 60, 90, 120, 180, 240, and 300 minutes after meal) were measured via an enzymatic assay (Wako Diagnostics, Richmond, VA). The total plasma sRAGE (basal and 240 minutes after meal) was measured with an ELISA (DRG00, R&D Systems, Minneapolis, MN, USA). This total sRAGE quantification approach captures the cRAGE and esRAGE isoforms. To quantify plasma esRAGE (basal and 240 minutes after meal), another ELISA was performed (K1009-1AS, One International, Mountain View, CA). The plasma cRAGE was determined by subtracting the esRAGE from the total sRAGE.^36-40^ Plasma inflammatory targets (fractalkine, interferon gamma, IL10, IL17A, IL1*β*, IL6, IL8, macrophage inflammatory protein 1 alpha [MIP1*α*], and tumor necrosis factor alpha [TNF*α*]) were assessed via the Milliplex MAP Human High Sensitivity magnetic bead panel (HSTCMAG-28SK, Millipore Corporation, Billerica, MA) in the basal state and 240 minutes after the meal challenge. Antibody beads, controls, buffers, serum matrix, and standards were prepared, and the kit was run as per manufacturers’ guidelines.

Markers of protein oxidation (OX) (methionine sulfoxide and aminoadipic acid, AAA) and AGE-free adducts (N*ε*-carboxymethyllysine, CML; N*ε*-carboxyethyllysine, CEL; 3-deoxyglucosone hydroimidazolone, 3DG-H; glyoxal hydroimidazolone-1, GH-1; and methylglyoxal hydroimidazolone-1, MG-H1) were measured in plasma samples obtained in the basal state and 240 minutes after the mixed-meal by liquid chromatography–tandem mass spectrometry as previously described.^38,40,41^ Briefly, plasma ultrafiltrate (10KD cutoff filter; Amicon, Millipore; Burlington, MA) was separated with a mobile phase gradient of methanol and water containing 0.20% heptafluorbutyric acid and quantified in a blinded fashion using stable internal isotope standards as described previously.^38,40,41^

### Skeletal Muscle Analyses

RAGE, Toll-like receptor 4 (TLR4), TIR-domain–containing adapter-inducing interferon-*β* (TRIF), total I*κ*B*α*, phosphorylated I*κ*B*α* (pI*κ*B*α*), and oligosaccharyltransferase subunit 48 (OST48) were measured by Western blot as previously described.^38^ Approximately 10-15 mg (wet weight) of frozen muscle tissue was homogenized by ceramic beads (Lysing Matrix D, FastPrep-24 homogenizer, MP Biomedical, Santa Ana, CA, USA) in 20 volumes of an ice-cold buffer made with 150 nM NaCL, 1 mM Na_2_ ethylenediaminetetraacetic acid, 1 mM ethylene glycol tetraacetic acid, 2.5 mM NA pyrophosphate, 1 mM *β*-glycerophosphate, 1 mM Na_3_VO_4_, 1% Triton, and 1 *μ*g•ml^−1^ leupeptin (Cell Signaling Technology, Beverly, MA) with an added 1X protease and phosphatase inhibitor cocktail (5872S, Cell Signaling Technology). Total protein concentration was determined via bicinchoninic acid assay (Pierce Biotechnology, Rockford, IL). Equal protein was loaded on a gradient gel (BioRad, Hercules, CA) and resolved using sodium dodecyl sulphate–polyacrylamide gel electrophoresis, transferred to a nitrocellulose membrane, and blocked with Odyssey Blocking Buffer (LI-COR Biosciences, Lincoln, NE) in tris-buffered saline for 1 hour at room temperature. RAGE (1:500, ab3611, Abcam, Cambridge, MA), TLR4 (1:2000, sc293072, Santa Cruz, Santa Cruz, CA), TRIF (1:1000, ab180689, Abcam), pI*κ*B*α* (1:1000, 9246, Cell Signaling), total I*κ*B*α* (1:1000, 9242, Cell Signaling), and OST48 (1:1000, sc74408, Santa Cruz) primary antibody incubations took place overnight, rocking, at 4°C. Secondary antibody (TLR4: 926-68072; TRIF, RAGE, and I*κ*B*α*: 925-68071; OST48 and pI*κ*B*α*: 926-32212; 1:20000, LICOR Biosciences) incubations occurred for 1 hour at room temperature while rocking. Protein expression (RAGE, TLR4, TRIF, and OST48) was visualized with an NIR system (LICOR Biosciences), normalized to total protein (Ponceau, Sigma Aldrich, St. Louis, MO), and quantified using Image Studio (LICOR Biosciences). Total I*κ*B*α* and pI*κ*B*α* were also visualized with the NIR system and quantified using Image Studio, but pI*κ*B*α* was normalized to total I*κ*B*α*. The RAGE was detected as two distinct bands (native: 43 kDa; glycosylated: 48 kDa). Expression of both bands was summed to represent total RAGE expression (and normalized to total protein).^38^ Although postprandial samples were collected in the study,^22^ because of tissue collection limitations, muscle protein targets were only assessed in the basal state.

Muscle inflammatory targets (fractalkine, interferon gamma, IL10, IL17 A, IL1*β*, IL6, IL8, MIP1*α*, and TNF*α*) were determined by Milliplex magnetic bead assay as described previously. Skeletal muscle samples were homogenized by ceramic beads (Lysing Matrix D; MP Biomedical; Irvine, CA) in 20 volumes of ice-cold PBS supplemented with 1X protease and phosphatase inhibitors (5872S, Cell Signaling Technology). After bead homogenization, samples were sonicated 2 × 30 seconds at 50% power on ice. Sonicated samples were then spun at 20000*g* for 30 minutes at 4°C, and the supernatant was retained for total protein concentration assessment via bicinchoninic acid assay (Pierce Biotechnology, Rockford, IL) and cytokine analyses. Antibody beads, controls, buffers, matrix (prepared in the muscle homogenization buffer), and standards were prepared, and the kit was run as per manufacturers’ guidelines.

### Statistical Analyses

Data were analyzed with GraphPad Prism (GraphPad Software, La Jolla, CA, USA) and evaluated for normality with a Shapiro-Wilk test. Non-normally distributed data were log-transformed before statistical analyses. A two-way analysis of variance with repeated measures was used to compare circulating OX markers, AGEs, inflammatory factors, and sRAGE concentrations between MHD and CON and time points (basal and 240 minutes after meal). Tukey’s post hoc test was used to examine specific differences when appropriate. Student’s t-test was used to compare subject characteristics, changes (absolute and percent) in circulating inflammatory concentrations, and basal inflammatory muscle protein expression between groups. Relationships between variables of interest were analyzed via Spearman’s rho correlation. Significance was set at *P* < .05, and a trend toward significance was recognized as *P* < .10. Data are presented as mean ± SE.

## Results

### Subject Characteristics

Subject characteristics are presented in Table 1 and have been previously reported.^22^ These data are presented here for context to the study’s main objective. MHD and CON participants were matched for age, sex, body mass index, and homeostatic model of assessment for insulin resistance and were therefore similar (*P* > .05). MHD participants underwent a dialysis period of 5 ± 1 years. In addition, 38% of MHD (>5 years) and 13% of CON (<5 years) participants reported a history of smoking. MHD exhibited higher circulating creatinine (+148%; *P* < .001), blood urea nitrogen (+109%; *P* < .001), and CRP (+85%; *P* = .01) but lower albumin (−8%; *P* = .04) and estimated glomerular filtrate rate (−160%; *P* < .001). There were no differences (*P* > .05) in basal nonesterified fatty acid concentrations (MHD: 0.64 ± 0.11; CON: 0.57 ± 0.06 mM) or area under the curve (MHD: 97.5 ± 14.3; CON: 100.1 ± 8.8 AU) from baseline to 300 minutes after meal between groups.

### Circulating Markers of Protein OX and AGEs

**Figure 1.**
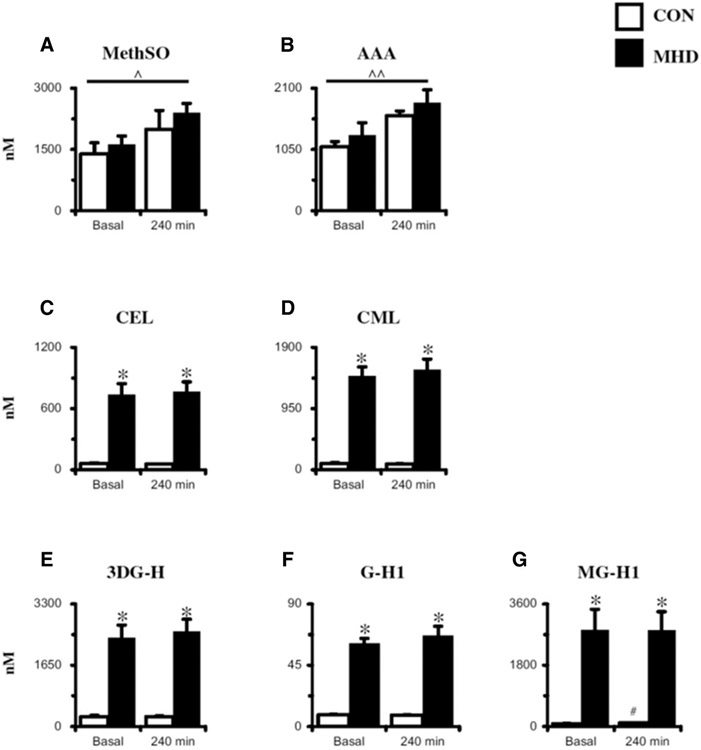
Plasma oxidation (panels A and B) and advanced glycation end products (panels C-G) in the basal state and after meal (+240 min) assessed via LC-MS/MS. See Mixed Meal Challenge in the Methods for more details regarding mixed meal nutrition information. 3DG-H, 3-deoxyglucosone hydroimidazolone; AAA, aminoadipic acid; CEL, N*ε*-carboxyethyl lysine; CML, N*ε*-carboxymethoyl lysine; CON, control subjects (n = 8); G-H1, glyoxal hydroimidazolone-1; LC-MS/MS, liquid chromatography–tandem mass spectrometry; MetSO, methionine sulfoxide; MG-H1, methylglyoxal hydroimidazolone-1; MHD, maintenance hemodialysis patients (n = 8). Data are mean ± SE; ^ Effect of time: *P* < .05; ^ Effect of time: *P* < .001; *P < .001 versus CON; #*P* < .05 versus Basal.

Plasma protein OX and AGEs in the basal and postprandial state are presented in Figure 1. Analysis of plasma OX yielded no basal differences (*P* > .05) in methionine sulfoxide or AAA between MHD and CON. An effect of time was observed for methionine sulfoxide (*P* = .03) and AAA (*P* <.001). For the circulating AGEs, basal concentrations were higher (*P* < .001) in MHD than those in CON (range = 7- to 343-fold greater). After the meal, CEL, CML, 3DG-H, and G-H1 remained unchanged (*P* > .05) in both groups, whereas MG-H1 increased 11.1-fold (*P* < .001) at 240 minutes in CON. Although MG-H1 increased in CON, concentrations remained ~28 fold lower than MHD at 240 minutes of the postprandial period.

### Circulating Markers of Inflammation

Basal total sRAGE (−32%), esRAGE (−53%), and cRAGE (−24%) were not statistically different (*P* > .05) between CON and MHD (Table 2). After the mixed meal, total sRAGE, esRAGE, and cRAGE remained unchanged (*P* > .05). However, independent of time, there was an effect of the group for the total sRAGE (*P* = .02) and esRAGE (*P* < .001) and a trend for the cRAGE (*P* = .09) to be higher in MHD than in CON.

**Figure 2.**
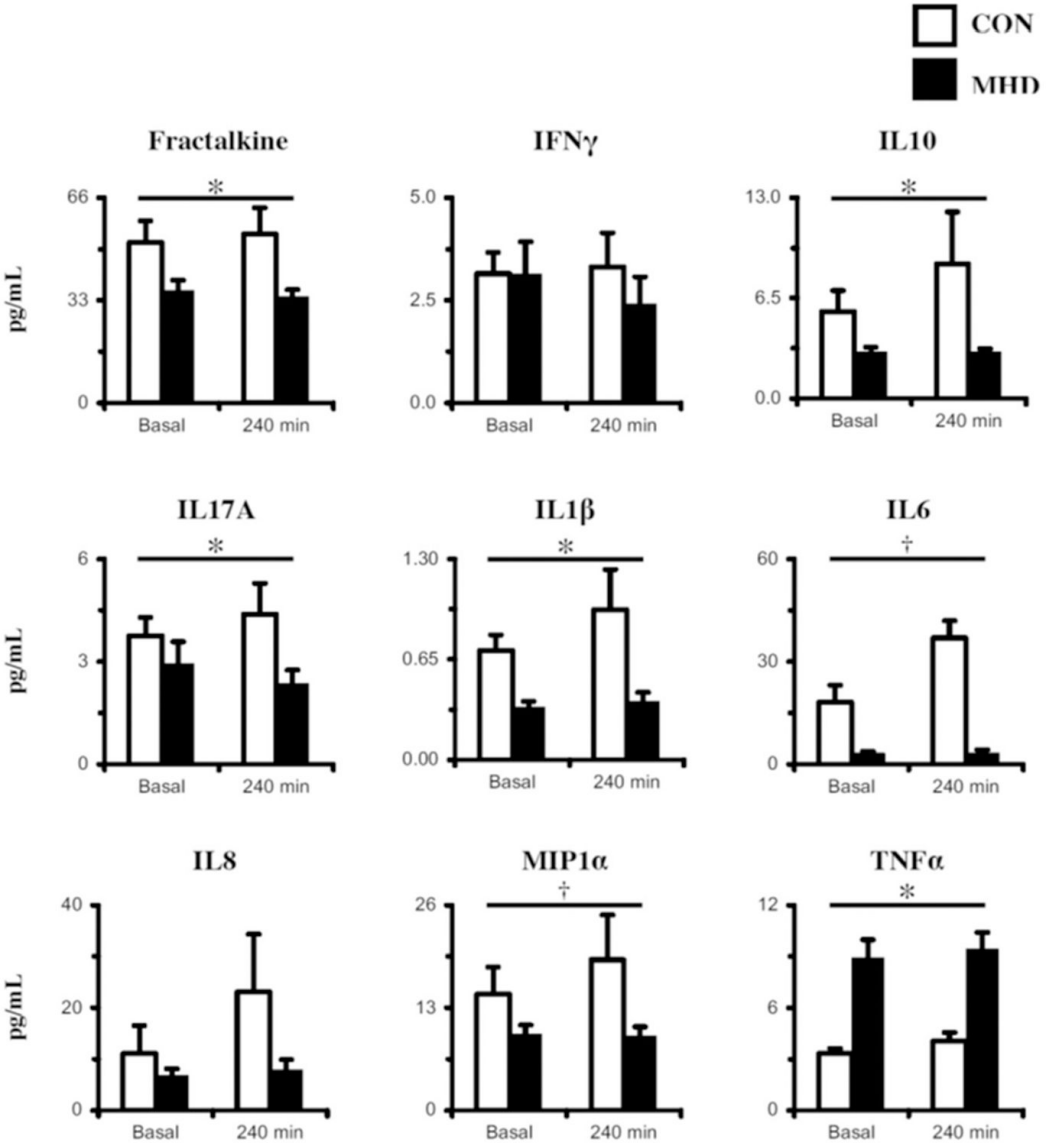
Plasma inflammatory biomarkers in the basal state and after meal (+240 min) assessed via multiplex. See Mixed Meal Challenge in the Methods for more details regarding mixed meal nutrition information. CON, control subjects (n = 8); IFN*γ*, interferon gamma; IL, interleukin; MHD, maintenance hemodialysis patients (n = 8); MIP1*α*, macrophage inflammatory protein 1 alpha; TNF*α*, tumor necrosis factor alpha; Data are mean ± SE; * Group effect: *P* < .05 versus CON; † Group effect: *P* < .10 versus CON.

Basal cytokine concentrations were similar (*P* > .05) between MHD and CON (Figure 2). After the mixed meal, all plasma inflammatory factor concentrations remained unchanged (*P* > .05). However, at both time points together (basal and 240 minutes after meal), there was a group effect (MHD < CON) for fractalkine (*P* = .003), IL10 (*P* = .007), IL17A (*P* = .04), and IL1*β* (*P* < .001) and a trend for IL6 (P = .06) and MIP1*α* (*P* = .07). In addition, there was an effect of the group (MHD > CON; *P* <.001) for TNF*α*.

### Muscle Protein Expression

**Figure 3.**
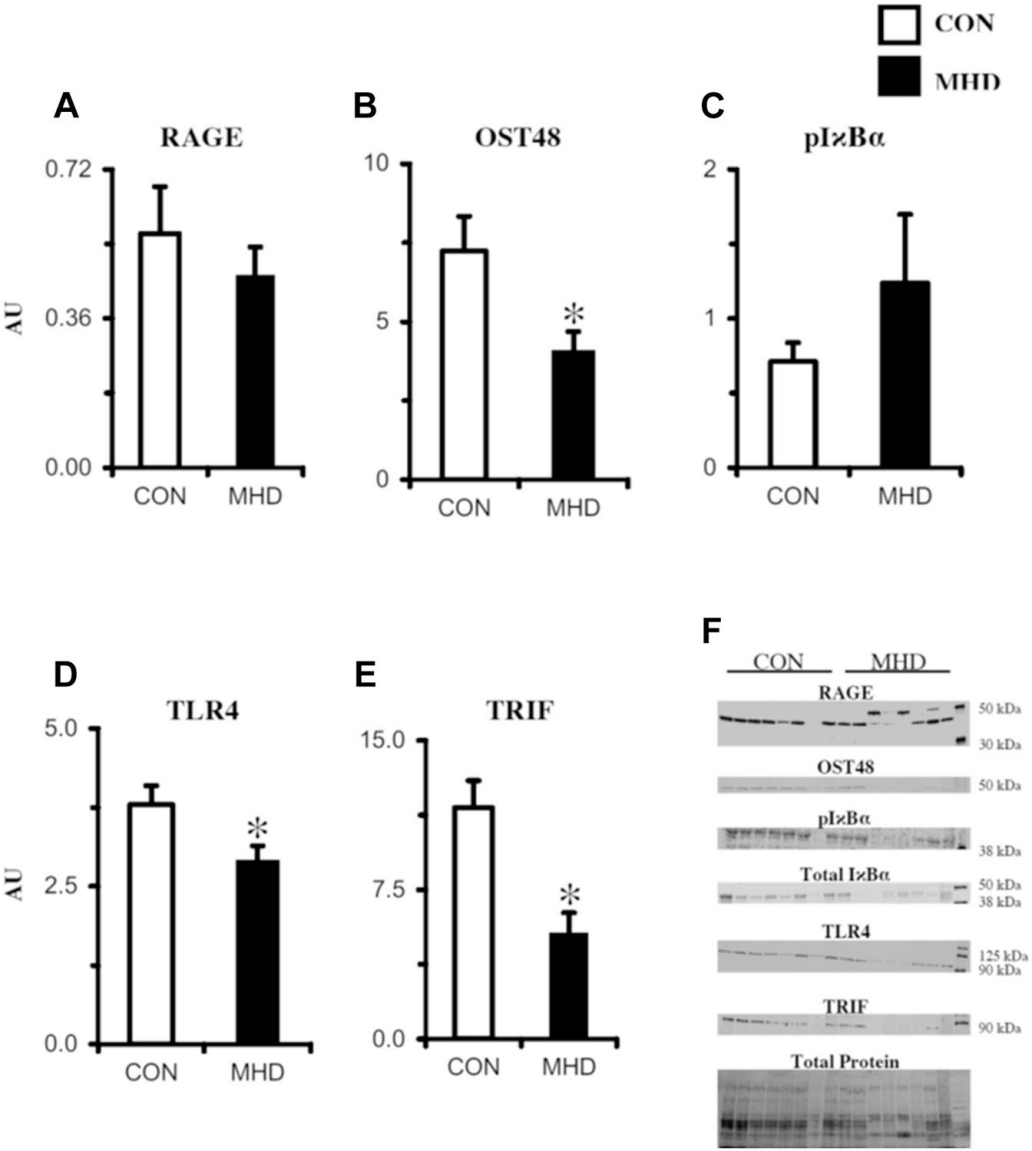
Biopsy-derived skeletal muscle (m. vastus lateralis) basal protein expression determined via Western blot of inflammatory receptors and downstream signaling components (A-E). Representative images of proteins assessed are depicted in panel F. CON, control subjects (n = 8); MHD, maintenance hemodialysis patients (n = 8); OST48, oligosaccharyltransferase subunit 48; pI*κ*B*α*, phosphorylated nuclear factor of kappa light polypeptide gene enhancer in B-cells inhibitor, alpha; RAGE, receptor for advanced glycation end products; TLR4, Toll-like receptor 4; TRIF, TIR-domain–containing adapter-inducing interferon-*β*. RAGE bands (43 and 48 kDa) were quantified together. Protein expression was normalized to total protein (A, B, D, and E) or total I*κ*B*α* (C). Individual data points were overlain on mean ± SE; *P < .05 versus CON.

**Figure 4.**
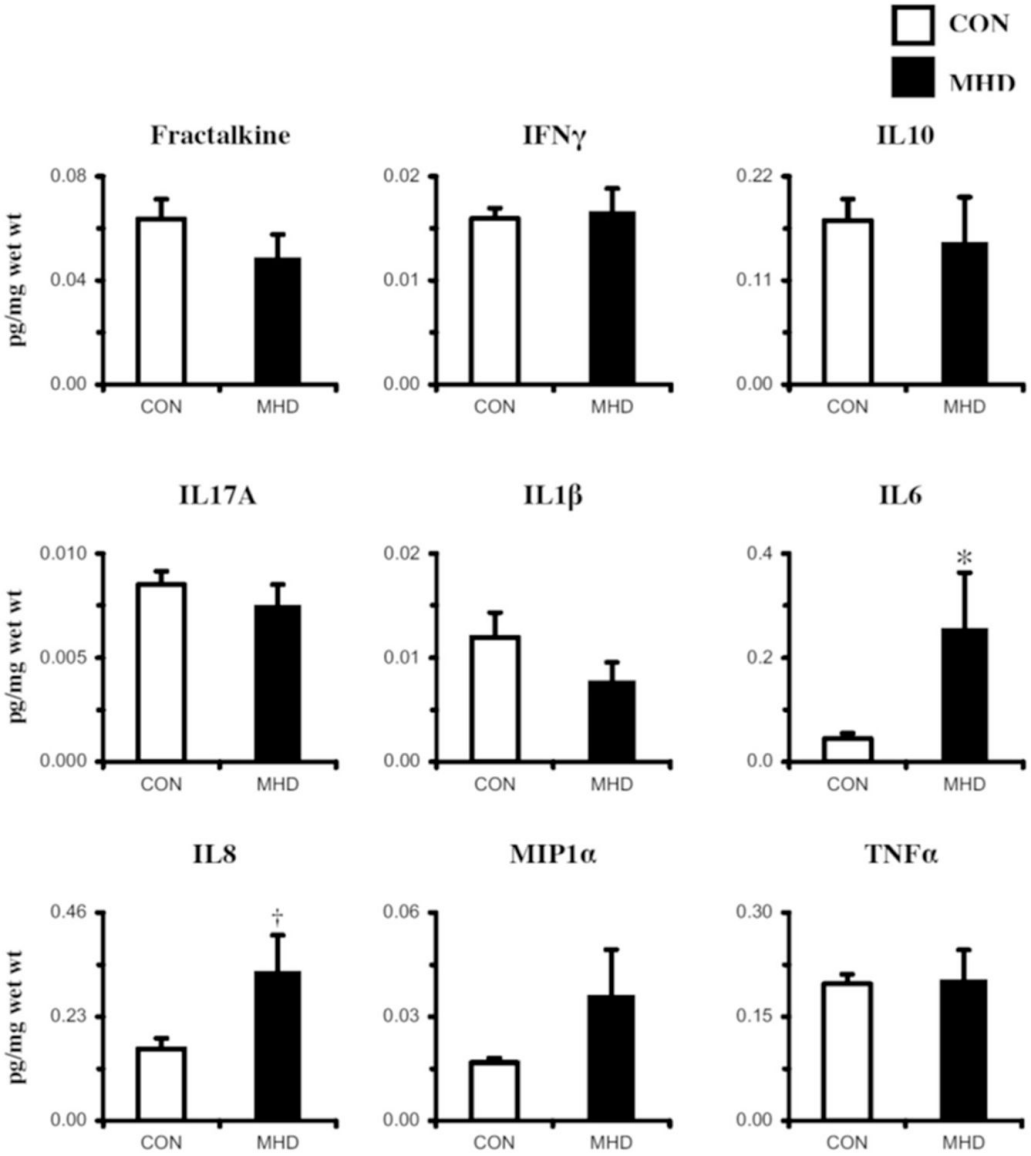
Biopsy-derived skeletal muscle (m. vastus lateralis) basal inflammatory cytokine protein expression determined via multiplex. Cytokine concentrations were normalized to mg muscle wet weight. CON, control subjects (n = 8); IFN*γ*, interferon gamma; IL, interleukin; MHD, maintenance hemodialysis patients (n = 8); MIP1*α*, macrophage inflammatory protein 1 alpha; TNF*α*, tumor necrosis factor alpha; Data are mean ± SE; * Group effect: *P* < .05 versus CON; †*P* < .10 versus CON.

Basal receptor signaling component protein expression in skeletal muscle of MHD and CON participants determined via Western blot is presented in Figure 3. Contrary to our original hypotheses, TLR4 (−27%; *P* = .03), TRIF (−74%; *P* = .002), and OST48 (−55%; *P* = .02) expression was lower in MHD than that in CON, whereas the RAGE and pI*κ*B*α*/total I*κ*B*α* were similar between groups (*P* > .05). To complement the inflammatory receptor protein expression, a targeted panel of nine inflammatory cytokines was also evaluated (Figure 4). Findings (pg target protein) were normalized in three ways: pg/mg muscle wet wt (Figure 4), pg/mL sample homogenate analyzed, and pg/*μ*g total protein. Subtle statistical differences were observed among the three normalization approaches (see Table S1 for pg/mL and pg/*μ*g protein normalizations). When cytokine protein was normalized to mg muscle wet weight, basal IL6 (+141%; *P* = .01) and IL8 (+70%; *P* = .08) were higher in MHD than those in CON, whereas the other cytokines were similar (*P* > .05).

### Relationships Among Variables of Interest

To explore potential relationships between variables, we conducted correlation analyses between clinical characteristics and biomarkers. Of note, for all participants in the basal state, we found an inverse relationship between body weight and plasma sRAGE (r = −0.52; *P* = .04) and esRAGE (r = −0.50; *P* = .05). As anticipated, basal plasma AGEs were strongly correlated with each other (r = 0.81-0.92; *P* < .001). Several basal plasma AGEs were inversely correlated with plasma IL10 (CML: r = −0.51, 3DG-H: r = −0.54, MG-H1: r = −0.55; *P* < .05) and positively correlated with plasma TNF*α* (CML: r = 0.86, 3DG-H: r = 0.80, CEL: r = 0.81, G-H1: r = 0.84, MG-H1: r = 0.70; *P* < .001) and muscle pg/*μ*g protein IL6 (CML: r = 0.60, 3DG-H: r = 0.65, CEL: r = 0.64, G-H1: r = 0.69, MG-H1: 0.66; *P* < .05). Basal plasma AGEs were also inversely correlated with muscle TRIF expression (r = −0.55-0.78; *P* < .05). Overall, few relationships were observed in response to the mixed meal challenge. Interestingly, when correlated with basal muscle myofibrillar synthesis rate (as reported in our previous publication),^22^ the myofibrillar synthesis rate was related to the percent change in plasma fractalkine (r = 0.56; *P* = .03), IL10 (r = 0.52; *P* = .04), IL1*β* (r = 0.55; *P* = .03), and TNF*α* (r = 0.53; *P* = .04).

## Discussion

The overarching goal of this investigation was to provide insight into the inflammatory phenotype in MHD by evaluating a comprehensive, yet targeted, group of circulating and skeletal muscle inflammatory biomarkers in the basal state and after a typical meal rich in protein recommended for this population. The principal findings from this study include the following: (1) circulating AGEs are substantially elevated in MHD patients compared with CON, (2) the basal circulating and skeletal muscle cytokine profile in MHD is generally proinflammatory, and (3) there was minimal effect of meal ingestion on modulating all inflammatory biomarkers in both groups.

A major objective of hemodialysis is to remove uremic solutes that exert toxic effects. Using the gold-standard quantification methods,^42^ we report that basal plasma AGE concentrations are 7- to 343-fold higher in the MHD patients than those in demographic controls. Noteworthy is that these assessments were made on samples from a nondialysis day (~24 hours after finishing dialysis), which suggests chronic AGE toxicity in MHD patients rather than only acutely during the dialysis period. Our results are consistent with previous reports,^4,43,44^ and note that MG-H1 is the most abundant AGE in MHD patients. Additional reports have observed a reduction in circulating AGEs in response to hemodialysis of up to 86%; however, circulating AGE burden remains substantially elevated compared with control subjects.^43-45^ Interestingly, the lack of correlation between reduction in AGE levels and AGE molecular size with dialysis^43^ highlights factors other than filter pore size govern AGE removal; thus, modified filtration approaches may need to be reconsidered.

AGEs exert many of their effects by activating RAGE signaling.^7,13-16^ Interestingly, RAGE expression was similar between groups, and OST48, TLR4, and TRIF expression was lower in MHD than that in the control group. This is intriguing and, to the best of our knowledge, the first report of RAGE protein expression in skeletal muscle of individuals undergoing MHD. In contrast to the findings presented here, one of the few studies conducted on circulating human cells demonstrated elevated RAGE protein expression in peripheral blood monocytes of dialysis patients,^46^ and another reported higher peripheral blood monocyte RAGE mRNA expression in patients with advanced CKD.^47^ Findings of high RAGE expression in circulating cells and similar RAGE expression in patients with renal disease suggest a potential tissue-specific effect. Interestingly, OST48 expression was lower in MHD. OST48 is a membrane-bound receptor that binds and transports AGEs from the extracellular to intracellular compartments for processing and removal.^48,49^ Therefore, OST48 may compete with RAGEs for ligands and likely exerts overall anti-inflammatory effects. In support, we observed a strong relationship between muscle OST48 and muscle IL10 (r = 0.59; *P* = .02). MHD muscle containing less OST48 than control subjects may be a fundamental component of the generally proinflammatory environment.

TLR4 is tasked with recognizing a host of ligands (some of which are shared with RAGEs) and signal to downstream pathway elements, including the TRIF adapter and the NF*κ*B pathway. Similar to our work presented here in muscle, Kuroki et al. found reduced TLR4 expression in circulating cells compared with controls, and dialysis duration (in years) was inversely related to TLR4 expression.^50^ Other studies have shown no difference^51^ or greater TLR4 expression in circulating cells of dialysis patients.^52^ To our knowledge, the only other study to examine TLR4 in the muscle of this patient population found higher TLR4 expression in dialysis patients than in a control group.^53^ The differential findings between our study and those by Verzola et al. may be explained by the analytical approach used to measure TLR4 (Western blot in our study vs. immunohistochemistry by Verzola et al.) or the type of dialysis technique used by the patients (i.e., MHD in our study and peritoneal dialysis by Verzola et al.). This is important to consider as hemodialysis is inflammatory in nature and has been shown to modulate genes involved with apoptosis and inflammation in skeletal muscle.^29^

The sRAGE is antagonistic to RAGE, exerting its effects by lowering inflammatory burden and improving indices of clinical health.^7,18,37,54-56^ Chronic sRAGE treatment in db/db mice reduces glomerulosclerosis and improves renal function.^54^ In humans, sRAGE is strongly associated with muscle mass,^56^ low sRAGE is related to the development of obesity and insulin resistance across the glucose tolerance continuum,^37^ and weight loss is related to increased sRAGE.^57^ In line with other studies on patients undergoing dialysis,^55,58^ here, we report elevated total sRAGE and esRAGE in MHD patients. One explanation for higher sRAGE in our MHD patients is the reliance on the kidney for sRAGE clearance, as total sRAGE concentration decreases after kidney transplantation.^59^ Another potential explanation for exaggerated sRAGE levels in clinical patients is this serves as a protective countermeasure to chronic inflammation. Therefore, in patients characterized by a proinflammatory environment, high sRAGE may be indicative of underlying pathologies. In fact, a very high sRAGE is associated with cardiovascular disease and all-cause mortality in clinical populations.^60^ It is clear that the sRAGE has protective effects; however, it remains to be seen if the sRAGE is upregulated as a compensatory mechanism to combat the inflammatory load in MHD or if impaired renal function blunts sRAGE clearance.

Patients undergoing dialysis exhibit a proinflammatory phenotype, reflective of a dynamic balance that has shifted away from factors that resolve (e.g., IL10) toward factors that perpetuate (e.g., TNF*α*) inflammation.^1-3,20,21,52,53^ Overall, here, we show the basal profile in MHD is proinflammatory. In the circulation, MHD had more proinflammatory CRP and TNF*α* and less anti-inflammatory IL10 than the control group. CRP is a well-studied inflammatory biomarker and is linked to negative forearm protein balance,^19^ often used as a proxy for skeletal muscle, and muscle loss^61^ in dialysis patients. CRP has also been shown to be localized to atherosclerotic lesions in humans, and CRP treatment of human monocytes leads to TNF*α* production.^62^ Interestingly, we found a strong correlation between circulating CRP and TNF*α* concentration (r = 0.70; *P* = .004). TNF*α* drives muscle wasting through its activation of the NF*κ*B pathway^63^ and caspase 3.^64^ Greater TNF*α* concentration in MHD muscle may explain the higher caspase 3 expression and hyperstimulation of myofibrillar protein synthesis rates in MHD reported in our previous publication.^22^

Provided muscle is a predominant metabolic tissue that produces and secretes inflammatory factors^65^ and experiences substantial wasting in renal failure,^66^ it is surprising that so little is known about inflammation concentrations in MHD muscle. In this study, we report a modest proinflammatory profile in MHD muscle as concentrations of the proinflammatory IL6 and chemotactic protein IL8 are elevated compared with the CON group. IL6 modulates its inflammatory effects in the muscle through protein balance disruption. In support, short-term IL6 infusion into healthy humans reduces muscle protein turnover by 50%,^67^ and IL6 injection into animals is reported to induce muscle wasting, and this effect is mediated by the suppressor of cytokine signaling 3.^68^ Suppressor of cytokine signaling 3 is generally considered proteolytic as it upregulates caspase 3. Therefore, elevated IL6 in MHD muscle may provide additional insight into greater caspase 3 protein content previously reported in this MHD cohort.^22^

In summary, this study provides a comprehensive, yet targeted, analysis of circulating and skeletal muscle inflammatory biomarkers in MHD patients in the basal state and after the ingestion of a meal rich in protein. Our findings of higher AGEs and proinflammatory cytokines and lower OST48 and anti-inflammatory cytokines add to the growing literature, indicating an overall proinflammatory environment in patients undergoing chronic MHD. Given the impact of renal replacement therapy on appetite and nutrient availability,^20,24-27^ we also sought to explore the biomarker effect of a mixed meal. In general, there was minimal effect of the meal on biomarkers explored, indicating a typical meal for this population does not exacerbate or attenuate the pre-existing inflammatory load. These findings suggest that the overall patient care may need to evolve to generate more specific and complementary therapeutic approaches in patients with CKD. The overarching goal should emphasize strategies that reduce the inflammatory load, including tailored physical activity and nutritional programs, thereby alleviating anabolic resistance.

### Practical Applications

In this study, we report an exaggerated circulating and skeletal muscle inflammatory environment, and a protein-rich meal did not appreciably affect inflammatory status. To curtail the loss in muscle mass MHD patients typically experience, these findings suggest dialysis treatments may need to evolve.

## Supplementary Material

Supplemental table 1

## Figures and Tables

**Table 1.** Clinical Characteristics of Control Subjects (CON) and Maintenance Hemodialysis Patients (MHD)

Characteristic	CON	MHD
M/F	6/2	6/2
Age (y)	50 ± 2	56 ± 5
Weight (kg)	94 ± 4	94 ± 9
BMI (kg/m^2^)	30.7 ± 1.3	32.3 ± 2.4
Fat (%)	29.4 ± 2.3	30.7 ± 3.5
HOMA-IR	4.7 ± 0.6	3.9 ± 0.9
BUN	15 ± 1	32 ± 3*
Albumin (g/dL)	3.9 ± 0.1	3.6 ± 0.1*
Creatinine (mg/dL)	1.1 ± 0.1	7.4 ± 0.9*
CRP (mg/L)	1.6 ± 4.2	11.9 ± 3.0*
eGFR (mL/min/1.73 m^2^)	82 ± 6	9 ± 1*

BMI, body mass index; BUN, blood urea nitrogen; CRP, c-reactive protein; eGFR, estimated glomerular filtrate rate; HOMA-IR, homeostatic model of assessment for insulin resistance.

Data are mean ± SE.

* *P* < .05 versus CON.

**Table 2.** Plasma Total sRAGE and sRAGE Isoforms in Control Subjects (CON) and Maintenance Hemodialysis Patients (MHD) in the Basal State and 240 Minutes After a Mixed Meal

Biomarker	CON (n = 8)		MHD (n = 8)	
	Basal (pg/mL)	240 min (pg/mL)	Basal (pg/mL)	240 min (pg/mL)
Total sRAGE*	932 ± 122	878 ± 120	1292 ± 152	1166 ± 115
esRAGE*	244 ± 37	239 ± 39	420 ± 53	414 ± 54
cRAGE†	688 ± 92	639 ± 86	873 ± 128	753 ± 90

cRAGE, cleaved receptor for advanced glycation end product; esRAGE, endogenous secretory receptor for advanced glycation end product; sRAGE, soluble receptor for advanced glycation end product.

Data are mean ± SE.

* Group effect: *P* < .05

† Group effect: *P* < .10.

## References

[1] Kalantar-Zadeh K, Stenvinkel P, Pillon L, Kopple JD. Inflammation and nutrition in renal insufficiency. Adv Ren Replace Ther. 2003;10:155–169. 14708070 10.1053/j.arrt.2003.08.008

[2] Kimmel PL, Phillips TM, Simmens SJ, Immunologic function and survival in hemodialysis patients. Kidney Int. 1998;54:236–244. 9648084 10.1046/j.1523-1755.1998.00981.x PMC6146918

[3] Carrero JJ, Chmielewski M, Axelsson J, Muscle atrophy, inflammation and clinical outcome in incident and prevalent dialysis patients. Clin Nutr. 2008;27:557–564. 18538898 10.1016/j.clnu.2008.04.007

[4] Thornalley PJ, Battah S, Ahmed N, Quantitative screening of advanced glycation endproducts in cellular and extracellular proteins by tandem mass spectrometry. Biochem J. 2003;375(Pt 3):581–592. 12885296 10.1042/BJ20030763 PMC1223712

[5] Bohlender JM, Franke S, Stein G, Wolf G. Advanced glycation end products and the kidney. Am J Physiol Ren. 2005;289:F645–F659. 10.1152/ajprenal.00398.2004 16159899

[6] Rodríguez-Ayala E, Anderstam B, Suliman ME, Enhanced RAGE-mediated NFkappaB stimulation in inflamed hemodialysis patients. Atherosclerosis. 2005;180:333–340. 15910860 10.1016/j.atherosclerosis.2004.12.007

[7] Gugliucci A, Menini T. The axis AGE-RAGE-soluble RAGE and oxidative stress in chronic kidney disease. Adv Exp Med Biol. 2014;824:191–208. 25039001 10.1007/978-3-319-07320-0_14

[8] Martens RJH, Broers NJH, Canaud B, Relations of advanced glycation endproducts and dicarbonyls with endothelial dysfunction and low-grade inflammation in individuals with end-stage renal disease in the transition to renal replacement therapy: a cross-sectional observational study. PLoS ONE. 2019;14:e0221058. 31408493 10.1371/journal.pone.0221058 PMC6692010

[9] Kratochvilová M, Zakiyanov O, Kalousová M, Kříha V, Zima T, Tesař V. Associations of serum levels of advanced glycation end products with nutrition markers and anemia in patients with chronic kidney disease. Ren Fal. 2011;33:131–137. 10.3109/0886022X.2010.541581 21332333

[10] Brownlee M. Advanced glycation end products in diabetic complications. Curr Opin Endocrinol Diabetes. 1996;3:291–297.

[11] Brownlee M. Biochemistry and molecular cell biology of diabetic complications. Nature. 2001;414:813–820. 11742414 10.1038/414813a

[12] Wautier J-L, Schmidt AM. Protein glycation: a firm link to endothelial cell dysfunction. Circ Res. 2004;95:233–238. 15297385 10.1161/01.RES.0000137876.28454.64

[13] Wautier MP, Chappey O, Corda S, Stern DM, Schmidt AM, Wautier JL. Activation of NADPH oxidase by AGE links oxidant stress to altered gene expression via RAGE. Am J Physiol Endorcinol Metab. 2001 ;280:E685–E694. 10.1152/ajpendo.2001.280.5.E685 11287350

[14] Yan SD, Schmidt AM, Anderson GM, Enhanced cellular oxidant stress by the interaction of advanced glycation end products with their receptors/binding proteins. J Biol Chem. 1994;269:9889–9897. 8144582

[15] Tanaka N, Yonekura H, Yamagishi S, Fujimori H, Yamamoto Y, Yamamoto H. The receptor for advanced glycation end products is induced by the glycation products themselves and tumor necrosis factor-alpha through nuclear factor-kappa B, and by 17beta-estradiol through Sp-1 in human vascular endothelial cells. J Biol Chem. 2000;275:25781–25790. 10829018 10.1074/jbc.M001235200

[16] Schmidt AM, Yan SD, Yan SF, Stern DM. The multiligand receptor RAGE as a progression factor amplifying immune and inflammatory responses. J Clin Invest. 2001;108:949–955. 11581294 10.1172/JCI14002 PMC200958

[17] Metz VV, Kojro E, Rat D, Postina R. Induction of RAGE shedding by activation of G protein-coupled receptors. PLoS ONE. 2012;7:e41823. 22860017 10.1371/journal.pone.0041823 PMC3408481

[18] Yonekura H, Yamamoto Y, Sakurai S, Novel splice variants of the receptor for advanced glycation end-products expressed in human vascular endothelial cells and pericytes, and their putative roles in diabetes-induced vascular injury. Biochem J. 2003;370(Pt 3):1097–1109. 12495433 10.1042/BJ20021371 PMC1223244

[19] Deger SM, Hung AM, Gamboa JL, Systemic inflammation is associated with exaggerated skeletal muscle protein catabolism in maintenance hemodialysis patients. J CI insight. 2017;2:299. 10.1172/jci.insight.95185 PMC5752392 29202452

[20] Wang XH, Mitch WE. Mechanisms of muscle wasting in chronic kidney disease. Nat Rev Nephrol. 2014;10:504–516. 24981816 10.1038/nrneph.2014.112 PMC4269363

[21] Garibotto G, Sofia A, Procopio V, Peripheral tissue release of interleukin-6 in patients with chronic kidney diseases: effects of end-stage renal disease and microinflammatory state. Kidney Int. 2006;70:384–390. 16760905 10.1038/sj.ki.5001570

[22] van Vliet S, Skinner SK, Beals JW, Dysregulated handling of dietary protein and muscle protein synthesis after mixed-meal ingestion in maintenance hemodialysis patients. Kidney Int Rep. 2018;3:1403–1415. 30450467 10.1016/j.ekir.2018.08.001 PMC6224635

[23] Draicchio F, van Vliet S, Ancu O, Integrin-associated ILK and PINCH1 protein content are reduced in skeletal muscle of maintenance haemodialysis patients. J Physiol. 2020;598:5701–5716. 32969494 10.1113/JP280441

[24] Cheung W, Yu PX, Little BM, Cone RD, Marks DL, Mak RH. Role of leptin and melanocortin signaling in uremia-associated cachexia. J Clin Invest. 2005;115:1659–1665. 15931394 10.1172/JCI22521 PMC1136984

[25] Kalantar-Zadeh K, Block G, McAllister CJ, Humphreys MH, Kopple JD. Appetite and inflammation, nutrition, anemia, and clinical outcome in hemodialysis patients. Am J Clin Nutr. 2004;80:299–307. 15277149 10.1093/ajcn/80.2.299

[26] Kalantar-Zadeh K, Fouque D. Nutritional management of chronic kidney disease. N Engl J Med. 2017;377:1765–1776. 29091561 10.1056/NEJMra1700312

[27] Sabatino A, Regolisti G, Karupaiah T, Protein-energy wasting and nutritional supplementation in patients with end-stage renal disease on hemodialysis. Clin Nutr. 2017;36:663–671. 27371993 10.1016/j.clnu.2016.06.007

[28] Ikizler TA, Pupim LB, Brouillette JR, Hemodialysis stimulates muscle and whole body protein loss and alters substrate oxidation. Am J Physiol Endorcinol Metab. 2002;282:E107–E116. 10.1152/ajpendo.2002.282.1.E107 11739090

[29] Shah V, Dominic E, Moseley P, Hemodialysis modulates gene expression profile in skeletal muscle. Am J Kid Dis. 2006;48:616–628. 16997058 10.1053/j.ajkd.2006.05.032

[30] Ikizler TA, Flakoll PJ, Parker RA, Hakim RM. Amino acid and albumin losses during hemodialysis. Kidney Int. 1994;46:830–837. 7996804 10.1038/ki.1994.339

[31] Foundation NK. Dietary guidelines for adults starting on hemodialysis, https://wwwkidneyorg/atoz/content/dietary_hemodialysis. Accessed August 1, 2019.

[32] Bergström J. Muscle electrolytes in man. Scand J Clin Lab Invest. 1962;14:1–110.

[33] Evans W, Phinney S, Young V. Suction applied to a muscle biopsy maximizes sample size. Med Sci Sports Exerc. 1982;14:101–102. 7070249

[34] Haus JM, Carrithers JA, Trappe SA, Trappe TA. Collagen, cross-linking, and advanced glycation end products in aging human skeletal muscle. J Appl Physiol. 2007;103:2068–2076. 17901242 10.1152/japplphysiol.00670.2007

[35] Mey JT, Blackburn B, Miranda E, Dicarbonyl stress and glyoxylase enzyme system regulation in human skeletal muscle. Am J Physiol Regul Integr Comp Physiol. 2017;314:R181–R190. 29046313 10.1152/ajpregu.00159.2017 PMC5867671

[36] Fuller KNZ, Valentine RJ, Miranda ER, Kumar P, Prabhakar BS, Haus JM. A single high-fat meal alters human soluble RAGE profiles and PBMC RAGE expression with no effect of prior aerobic exercise. Physiol Rep. 2018;6:e13811. 30047241 10.14814/phy2.13811 PMC6060105

[37] Miranda ER, Somal VS, Mey JT, Circulating soluble RAGE isoforms are attenuated in obese, impaired-glucose-tolerant individuals and are associated with the development of type 2 diabetes. Am J Physiol Endorcinol Metab. 2017;313:E631–E640. 10.1152/ajpendo.00146.2017 PMC5814601 28811295

[38] Perkins RK, Miranda ER, Karstoft K, Beisswenger PJ, Solomon TPJ, Haus JM. Experimental hyperglycemia alters circulating concentrations and renal clearance of oxidative and advanced glycation end products in healthy obese humans. Nutrients. 2019;11:532. 30823632 10.3390/nu11030532 PMC6471142

[39] Fuller KNZ, Miranda ER, Thyfault JP, Morris JK, Haus JM. Metabolic derangements contribute to reduced sRAGE isoforms in subjects with alzheimer’s disease. Mediators Inflamm. 2018;2018:1–10. 10.1155/2018/2061376 PMC5842684 29681765

[40] Miranda ER, Fuller KNZ, Perkins RK, Divergent changes in plasma AGEs and sRAGE isoforms following an overnight fast in T1DM. Nutrients. 2019;11:386. 30781793 10.3390/nu11020386 PMC6413006

[41] Beisswenger PJ, Howell SK, Russell G, Miller ME, Rich SS, Mauer M. Detection of diabetic nephropathy from advanced glycation endproducts (AGEs) differs in plasma and urine, and is dependent on the method of preparation. Amino Acids. 2014;46:311–319. 24036985 10.1007/s00726-013-1533-x

[42] Thornalley PJ. Measurement of protein glycation, glycated peptides, and glycation free adducts. Perit Dial Int. 2005;25:522–533. 16419322

[43] Agalou S, Ahmed N, Thornalley PJ, Dawnay A. Advanced glycation end product free adducts are cleared by dialysis. Ann N Y Acad Sci. 2005;1043:734–739. 16037300 10.1196/annals.1333.085

[44] Cornelis T, Eloot S, Vanholder R, Protein-bound uraemic toxins, dicarbonyl stress and advanced glycation end products in conventional and extended haemodialysis and haemodiafiltration. Nephrol Dial Transpl. 2015;30:1395–1402. 10.1093/ndt/gfv038 25862762

[45] Henle T, Deppisch R, Beck W, Hergesell O, Hänsch GM, Ritz E. Advanced glycated end-products (AGE) during haemodialysis treatment: discrepant results with different methodologies reflecting the heterogeneity of AGE compounds. Nephrol Dial Transpl. 1999;14:1968–1975. 10.1093/ndt/14.8.1968 10462279

[46] Hou FF, Ren H, Owen WF, Enhanced expression of receptor for advanced glycation end products in chronic kidney disease. J Am Soc Nephrol. 2004;15.1889–1896. 15213278 10.1097/01.asn.0000131526.99506.f7

[47] Linden E, Cai W, He JC, Endothelial dysfunction in patients with chronic kidney disease results from advanced glycation end products (AGE)-mediated inhibition of endothelial nitric oxide synthase through RAGE activation. Clin J Am Soc Nephrol. 2008;3:691–698. 18256374 10.2215/CJN.04291007 PMC2386710

[48] Li YM, Mitsuhashi T, Wojciechowicz D, Molecular identity and cellular distribution of advanced glycation endproduct receptors: relationship of p60 to OST-48 and p90 to 80K-H membrane proteins. Proc Natl Acad Sci U S A. 1996;93:11047–11052. 8855306 10.1073/pnas.93.20.11047 PMC38281

[49] Lu C, He JC, Cai W, Liu H, Zhu L, Vlassara H. Advanced glycation endproduct (AGE) receptor 1 is a negative regulator of the inflammatory response to AGE in mesangial cells. Proc Natl Acad Sci U S A. 2004;101:11767–11772. 15289604 10.1073/pnas.0401588101 PMC511050

[50] Kuroki Y, Tsuchida K, Go I, A study of innate immunity in patients with end-stage renal disease: special reference to toll-like receptor-2 and -4 expression in peripheral blood monocytes of hemodialysis patients. Int J Mol Med. 2007;19:783–790. 17390084

[51] Koc M, Toprak A, Arikan H, Toll-like receptor expression in monocytes in patients with chronic kidney disease and haemodialysis: relation with inflammation. Nephrol Dial Transpl. 2011;26:955–963. 10.1093/ndt/gfq500 20729266

[52] Grabulosa CC, Manfredi SR, Canziani ME, Chronic kidney disease induces inflammation by increasing Toll-like receptor-4, cytokine and cathelicidin expression in neutrophils and monocytes. Exp Cell Res. 2018;365:157–162. 29481790 10.1016/j.yexcr.2018.02.022

[53] Verzola D, Bonanni A, Sofia A, Toll-like receptor 4 signalling mediates inflammation in skeletal muscle of patients with chronic kidney disease. J Cachexia Sarcopenia Muscle. 2017;8:131–144. 27897392 10.1002/jcsm.12129 PMC5326826

[54] Wendt TM, Tanji N, Guo J, RAGE drives the development of glomerulosclerosis and implicates podocyte activation in the pathogenesis of diabetic nephropathy. Am J Pathol. 2003;162:1123–1137. 12651605 10.1016/S0002-9440(10)63909-0 PMC1851245

[55] Nishizawa Y, Koyama H. Endogenous secretory receptor for advanced glycation end-products and cardiovascular disease in end-stage renal disease.J Ren Nutr. 2008;18:76–82. 18089449 10.1053/j.jrn.2007.10.016

[56] Kim TN, Park MS, Lee EJ, The association of low muscle mass with soluble receptor for advanced glycation end products (sRAGE): the Korean Sarcopenic Obesity Study (KSOS). Diabetes Metab Res Rev. 2018;34. 10.1002/dmrr.2974 29271076

[57] Miranda ER, Fuller KNZ, Perkins RK, Endogenous secretory RAGE increases with improvements in body composition and is associated with markers of adipocyte health. Nutr Metab Cardiovasc Dis. 2018;28:1155–1165. 30297199 10.1016/j.numecd.2018.07.009 PMC6231965

[58] Kalousova M, Magdalena H, Kazderova M, Soluble receptor for advanced glycation end products in patients with decreased renal function. Am J Kid Dis. 2006;47:406–411. 16490618 10.1053/j.ajkd.2005.12.028

[59] Kalousová M, Bartošová K, Zima T, Skibová J, Teplan V, Viklický O. Pregnancy-associated plasma protein a and soluble receptor for advanced glycation end products after kidney transplantation. Kidney Blood Press Res. 2007;30:31–37. 17237617 10.1159/000098811

[60] Nin JWM, Jorsal A, Ferreira I, Higher plasma soluble Receptor for Advanced Glycation End Products (sRAGE) levels are associated with incident cardiovascular disease and all-cause mortality in type 1 diabetes: a 12-year follow-up study. Diabetes. 2010;59:2027–2032. 20522598 10.2337/db09-1509 PMC2911054

[61] Kaizu Y, Ohkawa S, Odamaki M, Association between inflammatory mediators and muscle mass in long-term hemodialysis patietns. Am J Kidney Dis. 2003;42:295–302. 12900811 10.1016/s0272-6386(03)00654-1

[62] Ballou SP, Lozanski G. Induction of inflammatory cytokine release from cultured human monocytes by C-reactive protein. Cytokine. 1992;4:361–368. 1420997 10.1016/1043-4666(92)90079-7

[63] Langen RC, Schols AM, Kelders MC, Wouters EF, Janssen-Heininger YM. Inflammatory cytokines inhibit myogenic differentiation through activation of nuclear factor-kappaB. FASEB J. 2001;15:1169–1180. 11344085 10.1096/fj.00-0463

[64] Geering B, Gurzeler U, Federzoni E, Kaufmann T, Simon H-U. A novel TNFR1-triggered apoptosis pathway mediated by class IA PI3Ks in neutrophils. Blood. 2011;117:5953–5962. 21478427 10.1182/blood-2010-11-322206

[65] Pedersen BK, Febbraio MA. Muscle as an Endocrine organ: Focus on muscle-derived interleukin-6. Physiol Rev. 2008;88:1379–1406. 18923185 10.1152/physrev.90100.2007

[66] Romagnani P, Remuzzi G, Glassock R, Chronic kidney disease. Nat Rev Dis Primers. 2017;3:17088. 29168475 10.1038/nrdp.2017.88

[67] van Hall G, Steensberg A, Fischer C, Interleukin-6 markedly decreases skeletal muscle protein turnover and increases nonmuscle amino acid utilization in healthy individuals. J Clin Endocrinol Metab. 2008;93:2851–2858. 18430776 10.1210/jc.2007-2223

[68] Zhang L, Du J, Hu Z, IL-6 and serum amyloid A synergy mediates angiotensin II-induced muscle wasting. J Am Soc Nephrol. 2009;20:604–612. 19158350 10.1681/ASN.2008060628 PMC2653674

