# Supplementary material for: Advanced Glycation End Products and Inflammatory Cytokine Profiles in Maintenance Hemodialysis Patients After the Ingestion of a Protein-Dense Meal
Source: J Ren Nutr. Author manuscript; Available in PMC 2023 Oct 17. (PMC10580815; doi:10.1053/j.jrn.2021.11.006)
Supplement: Supplemental table 1 [file NIHMS1934373-supplement-Supplemental_table_1.docx]

| **Supplemental Table 1.** Basal muscle protein inflammatory cytokine expression. | | |
| --- | --- | --- |
| **Biomarker** | **CON (n=8)** | **MHD (n=8)** |
| ***pg/mL*** |  |  |
| Fractalkine | 3.18±.38 | 2.19±.35 ^†^ |
| IFNγ | 0.80±0.05 | 0.73±0.02 |
| IL10 | 8.68±1.12 | 6.04±0.95 ^†^ |
| IL17A | 0.43±0.03 | 0.33±0.01 * |
| IL1β | 0.60±0.12 | 0.34±0.06 ^†^ |
| IL6 | 2.23±0.51 | 11.58±4.57 * |
| IL8 | 7.95±1.16 | 14.19±2.58 ^†^ |
| MIP1α | 0.84±0.06 | 1.47±0.40 |
| TNFα | 9.86±0.68 | 8.63±0.90 |
| ***pg/μg protein*** |  |  |
| Fractalkine | 0.001±0.0001 | 0.0008±0.0001 |
| IFNγ | 0.016±0.001 | 0.017±0.002 |
| IL10 | 0.0027±0.0003 | 0.0022±0.0003 |
| IL17A | 0.0001±0.00001 | 0.0001±0.00001 |
| IL1β | 0.0002±0.00003 | 0.0001±0.00002 |
| IL6 | 0.0008±0.0002 | 0.0037±0.0013 * |
| IL8 | 0.0025±0.0004 | 0.0051±0.0010 * |
| MIP1α | 0.0003±0.00002 | 0.0005±0.00013 |
| TNFα | 0.0031±0.0003 | 0.0033±0.0005 |
| Data are mean ± SE. Muscle cytokine expression was compared to known standards and concentrations were normalized to volume (mL) and μg protein. CON, control subjects; MHD, maintenance hemodialysis patients; IFNγ, interferon gamma; IL, interleukin; MIP1α, macrophage inflammatory protein 1 alpha; TNFα, tumor necrosis factor alpha * *P* < 0.05 vs CON; ^†^ *P* < 0.10 vs CON. | | |
